# Psychometric Validation of the Bangla Fear of COVID-19 Scale: Confirmatory Factor Analysis and Rasch Analysis

**DOI:** 10.1007/s11469-020-00289-x

**Published:** 2020-05-11

**Authors:** Najmuj Sakib, A. K. M. Israfil Bhuiyan, Sahadat Hossain, Firoj Al Mamun, Ismail Hosen, Abu Hasnat Abdullah, Md. Abedin Sarker, Mohammad Sarif Mohiuddin, Istihak Rayhan, Moazzem Hossain, Md. Tajuddin Sikder, David Gozal, Mohammad Muhit, S. M. Shariful Islam, Mark D. Griffiths, Amir H. Pakpour, Mohammed A. Mamun

**Affiliations:** 1 Undergraduate Research Organization, Gerua Road, Savar, Dhaka 1342 Bangladesh; 2 Department of Microbiology, Jashore University of Science and Technology, Jashore, Bangladesh; 3 Department of Public Health & Informatics, Jahangirnagar University, Savar, Dhaka Bangladesh; 4 Division of Diabetes, Department of Internal Medicine, Aichi Medical University, Nagakute, Aichi Japan; 5 Department of Economics, Bangabandhu Sheikh Mujibur Rahman Science and Technology University, Gopalganj, Bangladesh; 6 Institute of Allergy and Clinical Immunology of Bangladesh, Savar, Dhaka Bangladesh; 7 Department of Child Health and the Child Health Research Institute, The University of Missouri School of Medicine, Columbia, MO USA; 8 Asian Institute of Disability and Development, University of South Asia, Dhaka, Bangladesh; 9 CSF Global, Dhaka, Bangladesh; 10 Institute for Physical Activity and Nutrition, School of Exercise and Nutrition Sciences, Deakin University, Melbourne, Australia; 11 International Gaming Research Unit, Psychology Department, Nottingham Trent University, 50 Shakespeare Street, Nottingham, NG1 4FQ UK; 12 Department of Nursing, School of Health and Welfare, Jönköping University, Jönköping, Sweden

**Keywords:** COVID-19, Coronavirus, COVID-19 fear, FCV-19S Bangla, Fear of COVID-19 Scale, Bangladesh

## Abstract

The recently developed Fear of COVID-19 Scale (FCV-19S) is a seven-item uni-dimensional scale that assesses the severity of fears of COVID-19. Given the rapid increase of COVID-19 cases in Bangladesh, we aimed to translate and validate the FCV-19S in Bangla. The forward-backward translation method was used to translate the English version of the questionnaire into Bangla. The reliability and validity properties of the Bangla FCV-19S were rigorously psychometrically evaluated (utilizing both confirmatory factor analysis and Rasch analysis) in relation to socio-demographic variables, national lockdown variables, and response to the Bangla Health Patient Questionnaire. The sample comprised 8550 Bangladeshi participants. The Cronbach *α* value for the Bangla FCV-19S was 0.871 indicating very good internal reliability. The results of the confirmatory factor analysis showed that the uni-dimensional factor structure of the FCV-19S fitted well with the data. The FCV-19S was significantly correlated with the nine-item Bangla Patient Health Questionnaire (PHQ-90) (*r* = 0.406, *p* < 0.001). FCV-19S scores were significantly associated with higher worries concerning lockdown. Measurement invariance of the FCV-19S showed no differences with respect to age or gender. The Bangla version of FCV-19S is a valid and reliable tool with robust psychometric properties which will be useful for researchers carrying out studies among the Bangla speaking population in assessing the psychological impact of fear from COVID-19 infection during this pandemic.

The novel coronavirus 2019, now called severe acute respiratory syndrome coronavirus 2 (SARS-CoV-2) (causing the disease COVID-19), has currently expanded and shifted from an epidemic to global pandemic with emerging clinical outcomes (Guan et al. 2020; Huang et al. 2020). As of April 11 (2020), 210 countries or territories had confirmed cases of COVID-19 (World Health Organization 2020). To date, the scientific community has described the clinical course of COVID-19, counted cases and monitored its spread country-by-country, and has been trying to develop a vaccine (Dong et al. 2020; Wang et al. 2020). However, COVID-19 has a very high infection rate and relatively high mortality rate (i.e., 3.6% and 1.5% in and outside of China respectively; Baud et al. 2020); individuals are quite naturally afraid of getting the virus. Additionally, death toll overestimation (Roussel et al. 2020) and pandemic-related issues such as social distancing, quarantine, and isolation have amplified fears leading to stigma in several cases (Lin 2020).

Excessive fear alongside the social and economic consequences has the capacity to impair individuals’ rational thinking behavior and may lead to mental health suffering and psychosocial challenges (Ahorsu et al. 2020; Pakpour and Griffiths 2020; Pappas et al. 2009; Xiang et al. 2020). Moreover, mental health issues (i.e., depression, stress, panic, distress, etc.) can in more extreme cases result in suicidal ideation, suicide attempts, and actual suicide occurrence (Goyal et al. 2020; Mamun and Griffiths 2020a). Indeed, Mamun and Griffiths (2020b) recently reported the first case of suicide due to the fear of COVID-19 in Bangladesh. With a mortality rate of 6.2% in Bangladesh, the number of COVID-19 cases has been rising with individuals aged between 31 and 40 years being most infected (i.e., around 22%; Institute of Epidemiology, Disease Control and Research 2020). The rapid spread of COVID-19 appears to be creating a great concern in Bangladesh and the country has had many previous instances of disaster-related psychological suffering (Mamun and Griffiths 2020c; Mamun et al. 2019).

Separating facts from fear is vital because scientific information and data related to a disease outbreak change very frequently (Wenzel and Edmond 2003). Additionally, fear itself can be contagious as was demonstrated in 1994 when hundreds of thousands of people fled the Indian city of Surat to escape pneumonic plague, even though no cases had been confirmed based on the World Health Organization criteria (Epstein 2009). Consequently, reducing fear and discrimination among individuals can be important in controlling transmission.

Social marginalization and stigmatization deriving from fear in a disease outbreak may cause people to refute early symptoms that are clinically relevant, and may contribute to the disease remaining undetected in the society (Person et al. 2004). Likewise, there appears to be an epidemic of fear and aversion concerning the community transmission of COVID-19. For instance, local residents from one area protested against the burial of bodies who had died from COVID-19 in their community graveyard (Kamal 2020).

Based on these contextual concerns, Ahorsu et al. (2020) recently developed the Fear of COVID-19 Scale (FCV-19S) so that researchers could assess the fear of COVID-19 among individuals in their countries and communities. The seven-item FCV-19S is a quick and easy-to-use tool that underwent rigorous psychometric testing and appears to be suitable for both genders and all ages. As suggested by Mamun and Griffiths (2020b), there is an urgent need to carry out an epidemiological study nationwide in Bangladesh concerning the relationship between individuals’ mental health status (e.g., depression, anxiety, stress) and the fear of COVID-19. Therefore, the present study translated and validated the Bangla FCV-19S, and carried out factor analysis, as well as assessing its reliability and validity.

## Methods

### Participants and Procedure

The present cross-sectional study was the part of the Undergraduate Research Organization COVID-19 Project, which was carried out between April 1 and April 10, 2020. The target population was the general Bangladeshi population aged 10 years and older and being able to understand spoken Bangla. An online-based survey was developed and participants were recruited via social media (e.g., Facebook) and online blogs. The final sample comprised 8550 participants which far exceeded the recommended 5:1 ratio of number of participants to number of items (Floyd and Widaman 1995).

### Ethics

The study adhered to the guidelines of the Helsinki Declaration, 1975. The study was also approved by the Institute of Allergy and Clinical Immunology of Bangladesh ethics board (i.e., IRBIACIB/CEC/03202005). All participants provided informed consent. Parents provided informed consent for children and adolescent participants (*n* = 124, 1.45%). All participants were ensured concerning the anonymity and confidentiality of their data, and were provided with information about the nature and purpose of the study and the procedure, and were informed about their right to retract their data at any time.

### Adaptation of FCV-19S into Bangla

The forward-backward translation method was applied to adapt the FCV-19S into Bangla following previous studies (Arafat et al. 2016; Beaton et al. 2000; Sousa and Rojjanasrirat 2011). Two independent translators, one subject matter expert (a psychologist) and other experienced in culture and linguistic distinctions in English and Bangla, translated the seven-item scale into Bangla (i.e., forward translation). Both the versions were compiled and further translated back into English by another professional translator with medical translation proficiency and by one bilingual individual who had not seen the English version of the FCV-19S (i.e., back translation). After compiling the back translated versions, all were compared and submitted to an expert panel of five members. The panel scrutinized and finalized the items and all seven questions were retained. Pretesting was conducted among 140 individuals using online platforms across different age groups. The suggested changes were made by the research team. The final version of the questionnaire was then administrated on the large-scale study.

### Measures

#### Demographic Information

A contextual information sheet was used to obtain demographic and other information of the participants. The questions were related to (i) age, (ii) gender, (iii) educational status, (iv) occupational status, (v) current place of residence, (vi) marital status, (vii) whether they were a current smoker, (viii) whether they were a current alcohol consumer, and (ix) current health status.

#### Lockdown-Related Questions

To examine out if lockdown-related factors had any influence on participant’s fear, participants were asked: “If this lockdown situation persists more than a month, do you think you will have enough food supply?” “If you are a wage earner, do you think you may lose your job and/or face economic hardship in business?” and “Are you afraid of any probable economic recession due to this pandemic?” One of three responses was required for these questions (i.e., “Agree,” “Disagree,” or “Undecided”).

#### Bangla Patient Health Questionnaire

Participants’ health was assessed using the nine-item Bangla Patient Health Questionnaire (Bangla PHQ-9 (Chowdhury et al. 2004); original version (Kroenke et al. 2001)). The screening tool is used widely in both non-psychiatric and clinical settings. Symptoms of depression such as depressed mood, sleeping problems, feeling tired, appetite changes, concentration problems, and suicidal thoughts are assessed based on the past 2 weeks (Kroenke et al. 2001). Items are responded to on a 4-point Likert scale (0 = *not at all*, 1 = *several days*, 2 = *more than half of the days*, and 3 = *nearly every day*) and scores range from 0 to 27 (Kroenke et al. 2001). Cronbach’s alpha in the present study was 0.83.

#### Bangla Fear of COVID-19 Scale

The Bangla Fear of COVID-19 Scale assesses fear towards COVID-19 and was adapted from the English version of the scale published in the original paper by Ahorsu et al. (2020). The screening tool consists of seven items (e.g., “I cannot sleep because I am worried about getting coronavirus-19”) with a five-item Likert point response from 1 (*strongly disagree*) to 5 (*strongly agree*) and its score range is 7 to 35. The higher the score indicates the greater the fear of cororonavirus-19 (Ahorsu et al. 2020). The psychometric properties of the Bangla FCV-19S are presented in the “Results” section.

### Data Analysis

Descriptive statistics were used to describe study participants’ characteristics. Continuous data were reported as means (and SDs) and categorical data were reported as frequencies and percentages. There are two approaches for assessing psychometric characteristics of a scale: classical test theory (CTT) and modern test theory. CTT has been widely used to assess psychometric properties of various self-reported measures, and assumes that the item responses of a scale should be summed to compute a score. This score is a class true score (a representation of assessed outcome: in this study, fear). However, CTT has some limitations including (i) assuming a linear relationship between latent variables and observed scores and (ii) being dependent on sample and group. However, modern test theory such as item response theory (IRT) assumes that the relationship between latent variables and observed scores is not necessarily linear (Wirth and Edwards 2007). Instead, it could vary from one person to another. Several statistics were performed to assess CTT including item ceiling and floor effects (the percentage of participants who obtained highest and lowest possible scores, respectively). Values lower than 50% are acceptable (Strober et al. 2013), internal consistency (Cronbach’s *α* > 0.7 is acceptable), corrected item-total correlation (values > 0.4 are acceptable) (Pakpour et al. 2014), average variance extracted (AVE; values > 0.5 indicate satisfactory convergent reliability), composite reliability (CR; values higher than 0.6 indicate acceptable reliability), standard error of measurement (smaller values indicating better reliability), and confirmatory factor analysis (CFA). The uni-dimensional factor stricture of the FCV-19S was evaluated using CFA with diagonally weighted least squares (WLSMV) estimator. Several indices were used to assess model fit including comparative fit index (CFI) and Tucker–Lewis index (TLI) > 0.9; root mean square error of approximation (RMSEA) < 0.08; weighted root mean square residual (WRMSR) < 1.0; and non-significant chi-square (Li et al. 2017; Wu et al. 2017).

Regarding the IRT, a Rasch partial credit model was used to assess item difficulty, uni-dimensionality, item validity, item and person separation reliability, and item and person separation index. Item difficulty was evaluated using logit (an interval scale) with higher scores indicating more difficult items. Information-weighted fit statistic (infit) mean square (MnSq), and outlier-sensitive fit statistic (outfit) MnSq were used to assess item validity. Values ranging from 0.5 to 1.5 are considered acceptable good fit. Values greater than 0.7 for both item and person separation reliability are considered to be acceptable. Item and person separation index values are considered to be acceptable if they are greater than 2.

The uni-dimensionality of the FCV-19S was further assessed by conducting a principal component analysis of the residuals (PCAR). The first residual factor (as representative of uni-dimensionality) should explain more than 50% of the variance and the eigenvalues of the residuals should not be greater than 2.0 (Linacre 2012). Differential item functioning (DIF) was used to assess whether FCV-19S items are invariant across age and gender groups. A DIF value of 0.5 or higher indicates substantial DIF (i.e., invariance) across two groups. The concurrent validity was assessed by correlation (Pearson’s coefficient) of the FCV-19S and Patient Health Questionnaire (PHQ-9). Descriptive statistics were analyzed using SPSS 24.0, CFA using MPLUS 8.0, and Rasch model using WINSTEPS 4.3.0.

## Results

Characteristics of participants are summarized in Table 1. The mean age of participants was 26.5 years (SD ± 9.1). Overall, 56.0% were males and 71.6% were single. Most of the participants (82.0%) were educated at tertiary level (Table 1). The mean, SD, skewness, and kurtosis of all items of the FCV-19S are shown in Table 2. All items had skewness and kurtosis values within the ± 2.0 range, confirming that they were normally distributed. Significant floor and ceiling effects were not observed for all items of the FCV-19S, indicating that the scale was able to detect a change of fear score (Table 2). The Cronbach *α* value for the Bangla FCV-19S was 0.871, indicating very good internal reliability. The corrected item-total correlations were all between 0.59 and 0.70 and positive (Table 3).

**Table 1:** Participants characteristics (*N* = 8550)

	Mean ± SD or *n* (%)
Age (years)	26.53 ± 9.09
Gender (male)	4790 (56.0%)
Educational status	
No formal education	140 (1.6%)
Primary school	111 (1.3%)
Secondary school	304 (3.6%)
Higher secondary level	980 (11.5%)
Tertiary education	7015 (82.0%)
Occupational status	
Unemployed	317 (3.7%)
Day-laborer	51 (0.6%)
Farmer	47 (0.5%)
Business	396 (4.6%)
Student	5094 (59.6%)
Government employee	499 (5.8%)
Private employee	1156 (13.5%)
Retired	70 (0.8%)
Housewife	550 (6.4%)
Others	370 (4.3%)
Residence	
Village	1872 (21.9%)
Sub-district town	1174 (13.7%)
District town	2003 (23.4%)
Divisional town	3501 (40.5%)
Marital status	
Single	6120 (71.6%)
Married	2320 (27.1%)
Divorced/widowed	110 (1.3%)
Currently smoker (yes)	1259 (14.7%)
Alcohol use (yes)	215 (2.5%)
Self-reported health status	
Very good	1324 (15.5%)
Good	4563 (53.4%)
Acceptable	2383 (27.9%)
Poor	262 (3.1%)
Very poor	18 (0.2%)

**Table 2:** Item properties of the FCV-19S

Item #	Mean (SD)	Skewness	Kurtosis	Floor	Ceiling
#1 I am most afraid of Corona	3.62 (1.04)	− 0.82	0.18	427 (5.0%)	1425 (16.7%)
#2 It makes me uncomfortable to think about Corona	3.52 (1.06)	− 0.77	− 0.14	470 (5.5%)	1125 (13.2%)
#3 My hands become clammy when \I think about Corona	2.49 (1.13)	0.46	− 0.68	1673 (19.6%)	398 (4.7%)
#4 I am afraid of losing my life because of Corona	2.93 (1.22)	− 0.09	− 1.16	1272 (14.9%)	670 (7.8%)
#5 When I watching news and stories about Corona on social media, I become nervous or anxious	3.53 (1.07)	− 0.98	0.14	594 (6.9%)	936 (10.9%)
#6 I cannot sleep because I’m worrying about getting Corona	2.41 (1.11)	0.60	− 0.54	1766 (20.7%)	342 (4.0%)
#7 My heart races or palpitates when I think about getting Corona	2.88 (1.24)	0.05	− 1.22	1231 (14.4%)	749 (8.8%)

**Table 3:** Psychometric properties of the FCV-19S in item level

Item no.	Analyses from classical test theory					Analyses from Rasch		
	Factor loading^a^	Item-total correlation	Infit MnSq	Outfit MnSq	Difficulty	Discrimination	DIF contrast across gender^cd^	DIF contrast across age^ce^
FCV19S1	0.77	0.65	0.99	0.99	− 1.04	0.94	− 0.10	− 0.03
FCV19S2	0.72	0.59	1.12	1.16	− 0.84	0.90	− 0.17	− 0.12
FCV19S3	0.80	0.68	0.82	0.84	1.0	1.07	0.08	− 0.09
FCV19S4	0.80	0.70	0.94	0.95	0.25	1.06	0.06	0.01
FCV19S5	0.77	0.65	0.93	0.87	− 0.86	1.17	0.08	− 0.01
FCV19S6	0.78	0.65	0.91	0.93	1.16	1.07	0.06	0.26
FCV19S7	0.72	0.61	1.20	1.26	0.33	0.84	− 0.05	0.16

^a^Based on confirmatory factor analysis

^c^DIF contrast > 0.5 indicates substantial DIF

^d^DIF contrast across gender = Difficulty for females − Difficulty for males

^e^DIF contrast across age categories = Difficulty for participants with older age (i.e., ≥ 26.53 years) − Difficulty for participants with younger age (i.e., < 26.53 years)

*MnSq*, mean square error; *DIF*, differential item functioning

The results of the CFA are reported in Table 3 and showed that the single-factor structure of the FCV-19S fitted well with the data (CFI = 0.964, TLI = 0.947, RMSEA = 0.071, and WRMSR = 0.889). Factor loadings from the model ranged from 0.72 to 0.80 and were statistically significant. The fit of the data for Rasch model was acceptable: log likelihood chi-square = 116,264.6277, df = 116,964, *p* = 0.9261, and root mean square standard error (RMSE) = 0.7228. The item separation reliability and index were 1.0 and 55.65, respectively. Moreover, the person separation reliability and index were 0.86 and 2.43, respectively. AVE and CR were higher than 0.58 and 0.89, respectively, suggesting an evidence of construct reliability. The results of the PCAR showed that the raw variance of the FCV-19S explained by the Rasch measure was 61.4%. The unexplained variance in the first contrast was 9.1% (1.64 eigenvalue units), and in the second contrast was 6.9% (1.26 eigenvalue units), indicating the evidence of uni-dimensionality.

The infit and outfit MnSq of all seven items were within the acceptable range (i.e., 0.5 to 21.5) (Table 4). Table 4 shows that item difficulty ranged from − 1.04 to 1.16 logits, with the most difficult item being Item 6 (“I cannot sleep because I’m worrying about getting coronavirus-19”) and the easiest item being item 1 was (“I am most afraid of coronavirus-19”). As to the measurement invariance, all items in the FCV-19S did not show DIF by age and gender subgroups of the participants. To determine whether there were differences between the gender group and fear scores, an independent *t* test was performed. The results showed that females reported significantly higher scores than males concerning fear of COVID-19 (mean = 22.75 [SD = 5.65] vs. mean = 20.29 [SD = 5.90]; *t* = − 19.46, *p* < 0.001). In contrast, age did not correlate significantly with total score of the FCV-19S (*r* = − 0.014, *p* = 0.186). The partial correlation coefficient showed that total score of the FCV-19S was significantly correlated with PHQ-9 (*r* = 0.406, *p* < 0.001). A possible association between fear and lockdown worries was analyzed using a multiple linear regression analysis with adjustments for age and gender as well as PHQ-9. The results showed that FCV-19S was significantly associated with higher worries concerning lockdown (standardized beta coefficient = 0.141, *p* < 0.001). In addition, higher scores on PHQ-9 were associated with higher worries concerning lockdown (standardized beta coefficient = 0.057, *p* < 0.001).

**Table 4:** Psychometric properties of the FCV-19S at scale level

Psychometric testing	Value	Suggested cutoff
Internal consistency (Cronbach’s *α*)	0.871	> 0.7
Confirmatory factor analysis		
*χ*^2^ (*df*)	554.75 (14)*	Non-significant
Comparative fit index	0.964	> 0.9
Tucker–Lewis index	0.947	> 0.9
Root mean square error of approximation	0.071	< 0.08
Weighted root mean square residual	0.889	< 1.0
Average Variance Extracted	0.58	> 0.5
Composite Reliability	0.89	> 0.6
Standard error of measurement	2.130	The smaller, the better
Item separation reliability from Rasch	1.00	> 0.7
Item separation index from Rasch	55.65	> 2
Person separation reliability from Rasch	0.86	> 0.7
Person separation index from Rasch	2.43	> 2
Test-retest reliability by Pearson correlation	0.87	> 0.4

*p < 0.001

## Discussion

The main aim of the present study was to evaluate the psychometric characteristics of the FCV-19S among the Bangladeshi community using both classic (i.e., confirmatory factor analysis [CFA]) and modern (i.e., Rasch analysis) psychometric evaluation methods. The study showed that the Bangladeshi version of the FCV-19S had (i) strong internal consistency (as demonstrated by the very good Cronbach’s alpha), (ii) acceptable construct validity (as demonstrated by CFA), (iii) confirmed uni-dimensional structure (as demonstrated by the CFA and Rasch analysis), (iv) good concurrent validity (as demonstrated by the significant positive correlation with depression scores on the Patient Health Questionnaire), (v) scale items that were invariant across age and gender groups (as demonstrated by the Rasch analysis), and (vi) good face validity (as demonstrated by the significant association between higher worries concerning lockdown and score on the FCV-19S).

In general, the results of the study were comparable with the original Iranian validation (Ahorsu et al. 2020) and a recent Italian validation study (Soraci et al. 2020). However, factor loadings of the Bangla version of the FCV-19S were greater than those of both Iranian and Italian versions. The potential reason could be due to mean age of the sample and large sample size of the present study (*N* = 8677; mean age = 26.53 years, SD = 9.09) compared with the studies in Iran (*N* = 717; mean age = 31.25 years, SD = 12.68) and Italy (*N* = 249, mean age = 34.5 years, SD = 12.21; predominantly female sample). However, it can be argued that when there is a global pandemic, people at all ages feel threatened and answer questions in a similar way. Furthermore, the results obtained from the DIF analysis showed that the Bangla version of the fear questionnaire could be used in the general population irrespective of their age. In the present study, women reported higher levels of fear of COVID-19 than men. Fear can be an emotional response to an external factor. Gender differences caused by a reaction to an external threatening agent are not new. Previous studies have shown that women report more fear of contamination and disgust sensitivity than males (e.g., Olatunji et al. 2005).

Assessing fear of COVID-19 can help to assess the mental health of general populations during the pandemic. Knowing such information can help in providing information to targeted specific populations so that they can perform preventive COVID-19 behaviors to help reduce fear levels (Pakpour and Griffiths 2020). Fear in human triggers reactions (mostly psychological) which help in preparing an individual to respond to a threatening agent (Ferraro and Grange 1987). As the results of the present study show, fear of COVID-19 was significantly associated with participants’ depression scores and their worries concerning lockdown.

The present study was similar to the results obtained from the previous Iranian and Italian validation studies (Ahorsu et al. 2020; Soraci et al. 2020); both of which included depression scales to test for concurrent and criterion validity. All three validation studies reported significant positive associations between depression and FCV-19S score. This is not surprising but due to the cross-sectional nature of all three studies, it is unclear as to whether being depressed heightens the fear concerning COVID-19 or whether the fear concerning COVID-19 heightens depression (or both). Longitudinal studies are needed to examine the direction of causality.

The large sample size, consideration of broad age group, and utilization of both classic and modern psychometric assessments are among the strengths of the present study. However, the present study has some limitations. First, fear in the present study was assessed using a self-reported measure that can be influenced by factors such as social desirability, memory recall, and other common method biases. Studies using other methodologies are recommended (e.g., in-depth qualitative interviews, diary studies). Second, although a large sample size was included in the present study, the sample was a convenience sample and was not necessarily representative of the general population of Bangladesh. Future studies using nationally representative studies are needed to confirm the results reported here. Third, the study design was cross-sectional, and therefore, the associations found between variables provide little insight into causality. Future research should include longitudinal designs to assess (as aforementioned) relationships between depression and fear of COVID-19. Finally, this study did not examine the stability of the FCV-19S over time. Future research should therefore incorporate test-retest reliability measures into the design of their studies. Overall, the results of the present study showed that the Bangla version of the FCV-19S has robust psychometric properties and can be used to assess fear of COVID-19 during the pandemic among the general Bangladeshi population.

## Data Availability

Data will be available on request.

## References

[CR1] Ahorsu, D. K., Lin, C.-Y., Imani, V., Saffari, M., Griffiths, M. D., & Pakpour, A. H. (2020). The fear of COVID-19 scale: Development and initial validation. *International Journal of Mental Health and Addiction*. Epub ahead of print. 10.1007/s11469-020-00270-8. 10.1007/s11469-020-00270-8 PMC7100496 32226353

[CR2] Arafat, S., Chowdhury, H., Qusar, M., & Hafez, M. (2016). Cross cultural adaptation and psychometric validation of research instruments: A methodological review. *Journal of Behavioral Health, 5*(3), 129–136.

[CR3] Baud, D., Qi, X., Nielsen-Saines, K., Musso, D., Pomar, L., & Favre, G. (2020). Real estimates of mortality following COVID-19 infection. *The Lancet Infectious Diseases*. Epub ahead of print. 10.1016/S1473-3099(20)30195-X. 10.1016/S1473-3099(20)30195-X PMC7118515 32171390

[CR4] Beaton DE, Bombardier C, Guillemin F, Ferraz MB (2000). Guidelines for the process of cross-cultural adaptation of self-report measures. Spine.

[CR5] Chowdhury AN, Ghosh S, Sanyal D (2004). Bangla adaptation of Brief Patient Health Questionnaire for screening depression at primary care. Journal of the Indian Medical Association.

[CR6] Dong L, Hu S, Gao J (2020). Discovering drugs to treat coronavirus disease 2019 (COVID-19). Drug Discoveries & Therapeutics.

[CR7] Epstein JM (2009). Modelling to contain pandemics. Nature.

[CR8] Ferraro KF, Grange RL (1987). The measurement of fear of crime. Sociological Inquiry.

[CR9] Floyd FJ, Widaman KF (1995). Factor analysis in the development and refinement of clinical assessment instruments. Psychological Assessment.

[CR10] Goyal K, Chauhan P, Chhikara K, Gupta P, Singh MP (2020). Fear of COVID 2019: first suicidal case in India. Asian Journal of Psychiatry.

[CR11] Guan, W. J., Ni, Z. Y., Hu, Y., Liang, W. H., Ou, C. Q., He, J. X., ... & Du, B. (2020). Clinical characteristics of coronavirus disease 2019 in China. *New England Journal of MedicineMedicine, 382*(18), 1708-1720. 10.1056/NEJMoa2002032 PMC7092819 32109013

[CR12] Huang, C., Wang, Y., Li, X., Ren, L., Zhao, J., Hu, Y., ... & Cheng, Z. (2020). Clinical features of patients infected with 2019 novel coronavirus in Wuhan. China. *The Lancet, 395*(10223), 497–506. 10.1016/S0140-6736(20)30183-5 PMC7159299 31986264

[CR13] Institute of Epidemiology, Disease Control and Research (2020). Covid-19 status Bangladesh. Retrieved May 5, 2020, from: https://www.iedcr.gov.bd/index.php/component/content/article/website/images/files/nCoV/Confirmed%20cases%20in%20Bangladesh_upto%20April%2011_2020.pdf.

[CR14] Kamal, R. S. (2020). Fear, hatred and stigmatization grip Bangladesh amid Covid-19 outbreak. The Business Standard, March 26. Retrieved May 5, 2020, from: April 13, 2020, from: https://tbsnews.net/thoughts/fear-hatred-and-stigmatization-grip-bangladesh-amid-covid-19-outbreak-61129.

[CR15] Kroenke, K., Spitzer, R. L., & Williams, J. B. W. (2001). The PHQ-9: Validity of a brief depression severity measure. *Journal of General Internal Medicine, 16*(9), 606–613. 10.1046/j.1525-1497.2001.016009606.x PMC1495268 11556941

[CR16] Lin C-Y (2020). Social reaction toward the 2019 novel coronavirus (COVID-19). Social Health and Behavior.

[CR17] Lin CY, Broström A, Nilsen P, Griffiths MD, Pakpour AH (2017). Psychometric validation of the Persian Bergen Social Media Addiction Scale using classic test theory and Rasch models. Journal of Behavioral Addictions.

[CR18] Linacre, J.M., 2012. A user’s guide to WINSTEPS® MINISTEP Rasch-Model Computer Programs Program Manual 3.80.0. Chicago, IL: Winsteps.

[CR19] Mamun MA, Griffiths MD (2020). A rare case of Bangladeshi student suicide by gunshot due to unusual multiple causalities. Asian Journal of Psychiatry.

[CR20] Mamun, M. A., & Griffiths, M. D. (2020b). First COVID-19 suicide case in Bangladesh due to fear of COVID-19 and xenophobia: Possible suicide prevention strategies. *Asian Journal of Psychiatry, 51*, 102073. 10.1016/j.ajp.2020.102073 PMC7139250 32278889

[CR21] Mamun, M. A., & Griffiths, M. D. (2020c). PTSD-related suicide six years after the Rana Plaza collapse in Bangladesh. *Psychiatry Research*, Epub ahead of print. 10.1016/j.psychres.2019.112645, 287, 112645. 10.1016/j.psychres.2019.112645 31685284

[CR22] Mamun, M. A., Huq, N., Papia, Z. F., Tasfina, S., & Gozal, D. (2019). Prevalence of depression among Bangladeshi village women subsequent to a natural disaster: A pilot study. *Psychiatry Research, 276*, 124–128. 10.1016/j.psychres.2019.05.007 31077883

[CR23] Olatunji BO, Sawchuk CN, Arrindell WA, Lohr JM (2005). Disgust sensitivity as a mediator of the sex differences in contamination fears. Personality and Individual Differences.

[CR24] Pakpour, A. H., & Griffiths, M. D. (2020). The fear of COVID-19 and its role in preventive behaviors. Journal of Concurrent Disorders, 2(1), 58–63

[CR25] Pakpour AH, Zeidi IM, Yekaninejad MS, Burri A (2014). Validation of a translated and culturally adapted Iranian version of the International Index of Erectile Function. Journal of Sex & Marital Therapy.

[CR26] Pappas G, Kiriaze IJ, Giannakis P, Falagas ME (2009). Psychosocial consequences of infectious diseases. Clinical Microbiology and Infection.

[CR27] Person, B., Sy, F., Holton, K., Govert, B., Liang, A., Garza, B., … Zauderer, L. (2004). Fear and stigma: The epidemic within the SARS outbreak. *Emerging Infectious Diseases, 10*, 358–363. 10.3201/eid1002.030750 PMC3322940 15030713

[CR28] Roussel, Y., Giraud-Gatineau, A., Jimeno, M.-T., Rolain, J.-M., Zandotti, C., Colson, P., & Raoult, D. (2020). SARS-CoV-2: Fear versus data. *International Journal of Antimicrobial Agents*. 10.1016/j.ijantimicag.2020.105947. 10.1016/j.ijantimicag.2020.105947 PMC7102597 32201354

[CR29] Soraci, P., Ferrari, A., Abbiati, F. A., Del Fante, E., De Pace, R., Urso, A., & Griffiths, M. D. (2020). Validation and psychometric evaluation of the Italian version of the Fear of COVID-19 Scale. *International Journal of Mental Health and Addiction*. Epub ahead of print. 10.1007/s11469-020-00277-1. 10.1007/s11469-020-00277-1 PMC7198091 32372892

[CR30] Sousa, V. D., & Rojjanasrirat, W. (2011). Translation, adaptation and validation of instruments or scales for use in cross-cultural health care research: A clear and user-friendly guideline. *Journal of Evaluation in Clinical Practice, 17*, 268–274. 10.1111/j.1365-2753.2010.01434.x 20874835

[CR31] Strober, B. E., Nyirady, J., Mallya, U. G., Guettner, A., Papavassilis, C., Gottlieb, A. B., ... & Lebwohl, M. (2013). Item-level psychometric properties for a new patient-reported psoriasis symptom diary. *Value in Health, 16*(6), 1014–1022. 10.1016/j.jval.2013.07.002 24041351

[CR32] Wang, D., Hu, B., Hu, C., Zhu, F., Liu, X., Zhang, J., ... & Zhao, Y. (2020). Clinical characteristics of 138 hospitalized patients with 2019 novel coronavirus-infected pneumonia in Wuhan, China. *JAMA, 323*(11), 1061–1069. 10.1001/jama.2020.1585 PMC7042881 32031570

[CR33] Wenzel RP, Edmond MB (2003). Managing SARS amidst uncertainty. New England Journal of Medicine.

[CR34] Wirth, R. J., &l Edwards, M. C. (2007). Item factor analysis: Current approaches and future directions. *Psychological Methods, 12*(1), 58–79. 10.1037/1082-989X.12.1.58 PMC3162326 17402812

[CR35] World Health Organization (2020). Coronavirus disease (COVID-2019): Situation report-82. Retrieved May 5, 2020, from: https://www.who.int/docs/default-source/coronaviruse/situation-reports/20200411-sitrep-82-covid-19.pdf?sfvrsn=74a5d15_2.

[CR36] Wu, T. Y., Lin, C. Y., Årestedt, K., Griffiths, M. D., Broström, A., & Pakpour, A. H. (2017). Psychometric validation of the Persian nine-item Internet Gaming Disorder Scale–Short Form: Does gender and hours spent online gaming affect the interpretations of item descriptions? *Journal of Behavioral Addictions, 6*(2), 256–263. 10.1556/2006.6.2017.025 PMC5520122 28571474

[CR37] Xiang YT, Yang Y, Li W, Zhang L, Zhang Q, Cheung T, Ng CH (2020). Timely mental health care for the 2019 novel coronavirus outbreak is urgently needed. The Lancet Psychiatry.

